# Integrated metabolomics and network pharmacology analysis to reveal the mechanisms of Wenshenyang decoction in the treatment of chronic kidney disease

**DOI:** 10.3389/fphar.2025.1500463

**Published:** 2025-04-30

**Authors:** Ge Jin, Zongjiang Zhao

**Affiliations:** School of Traditional Chinese Medicine, Beijing University of Chinese Medicine, Beijing, China

**Keywords:** chronic kidney disease, metabolomics, network pharmacology, molecular mechanisms, experimental verification

## Abstract

**Background:**

Wenshenyang decoction (WSY) has been shown to have a considerable effect on restoring renal function and improving kidney Yang deficiency syndrome in patients with CKD. However, its mechanism remains unclear.

**Aims:**

This study aimed to integrated metabolomics and network pharmacology analysis combined with *in vitro* experiments to reveal the mechanisms.

**Materials and methods:**

Patients were selected from a clinical trial. LC-MS (Liquid chromatography-mass spectrometry) was used to investigate the differential metabolites and pathways. Spearman correlation analysis was performed between differential metabolites and clinical phenotypes. “Drug-component-differential metabolite” network was constructed to predict the core components and hub genes, and validated by molecular docking. On this basis, the effects of core components of WSY on the viability of Human Kidney-2 cells (HK-2) induced by doxorubicin (DOX) was detected by CCK-8, and RT-qPCR (Reverse transcription quantitative polymerase chain reaction) was used to detect the mRNA expression level of hub genes and related targets.

**Key findings:**

LC-MS detected 54 differential metabolites, of which 35 metabolites showed up regulated, and 19 decreased. Spearman analysis showed that the differential metabolites were correlated with the clinical phenotype. KEGG (Kyoto Encyclopedia of Genes and Genomes) enrichment analysis showed that WSY mainly affected linoleic acid metabolism, FcεRI signaling pathway, and unsaturated fatty acid biosynthesis. The “Drug-component-differential metabolite” network showed that the core components of WSY were quercetin, luteolin and kaempferol, and the hub genes were PTGS2, AKT1, MMP9, EGFR and MMP2. Molecular docking showed that they had good biological binding capacity. *In vitro* cell experiments further showed that quercetin, luteolin and kaempferol could significantly activate the cells and reduce the mRNA levels of PTGS2, AKT1, MMP9, EGFR, MMP2, and ANGPTL4, and increase the level of FGFR1, SIRT3 and the glucocorticoid receptor (GR).

**Significance:**

WSY has multi-component and multi-target properties in the treatment of CKD kidney Yang deficiency syndrome, and its mechanism may be related to anti-inflammatory and anti-fibrotic effects. This study provides a methodological reference for the treatment of CKD.

## Graphical Abstract

**Figure:**
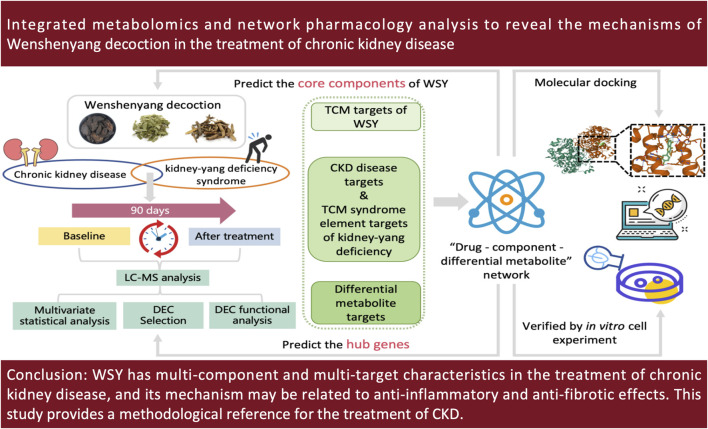

## Highlights

• WSY altered 54 different metabolites, of which 35 were upregulated and 19 were down-regulated.
• The core components of WSY were quercetin, luteolin, and kaempferol.
• Quercetin, luteolin and kaempferol primarily target PTGS2, AKT1, MMP9, EGFR and MMP2.
• The therapeutic mechanism of WSY for CKD may be associated with the synergistic anti-inflammatory and anti-fibrotic effects of its core components.

## 1 Introduction

Chronic kidney disease (CKD) is a chronic disease caused by structural and functional disorders of the kidneys (Li et al., 2023). The incidence and prevalence of CKD are increasing worldwide with the aggravation of social ageing (Indah Wardani et al., 2023). According to statistics (Maya-Quinta et al., 2023), there are currently about 850 million CKD patients worldwide, and it is expected to become the fifth most common disease affecting human life expectancy by 2040. In recent years, growing evidence suggests traditional Chinese medicine (TCM) has emerged as one of the most promising complementary methods for the prevention of CKD (Wang and Hu, 2017).

Wenshenyang decoction (WSY) is an herbal formula composed of *Cistanche deserticola* (Ma.), *Epimedium sagittatum* (Maxim.) and *Drynaria roosii* (Nakaike.) (http://mpns.kew.org *New version* January 2024). According to the 2020 edition of the Pharmacopoeia of the People’s Republic of China, the above herbal medicines have been suggested to be effective in tonifying the kidney yang, replenishing essential blood, and strengthening the muscles and bones. Previous animal studies have shown that WSY can improve symptoms such as oedema and fatigue in CKD kidney Yang deficiency model rats by regulating the TLR4/MyD88/NF-κB signalling pathway, AVP-V2R-AQP2 signalling pathway, and HIF1α/NLRP3/GSDMD signalling pathway (Zheng et al., 2021). However, these studies have primarily focused on a single target or pathway, and are insufficient to explain the overall mechanism of action of a complex TCM formula with multiple components and targets.

Metabolomics is a key technology for detecting and identifying small molecules produced by the human microbiome and understanding the functional roles of these microbial metabolites, which can be used to reveal key metabolites and their associations with disease (Costa-Lotufo et al., 2022). Network pharmacology is an effective tool for exploring the active ingredients of TCM from a holistic perspective. In recent years, more and more studies have revealed the molecular mechanism of TCM through network pharmacology combined with metabolite analysis. The combination of these two methods can break through the limitations of TCM, providing new perspectives and ideas for TCM research (Wang et al., 2024). In this study, we used a novel integrated strategy to explore the biological mechanisms of WSY therapy for CKD based on metabolomics and network pharmacology. First, metabolites biomarkers and pathways were analyzed by metabolomics and multivariate data analysis based on ultra-high performance liquid chromatography-tandem mass spectrometry (UPLC-MS/MS), and their correlation with clinical phenotype were analysed by spearman correlation analysis. Secondly, the core components and the hub genes of WSY for CKD treatment were predicted through network pharmacology. Finally, followed by molecular docking and *in vitro* experiments to verify the results. This study also provides a new idea for the systematic study of the effective ingredients of TCM.

## 2 Materials and methods

### 2.1 Clinical study

#### 2.1.1 Patients and interventions

Patients were selected from a randomized, controlled, double-blind clinical trial, in which a subset of typical patients were diagnosed by pathologic examination after renal biopsy. It was conducted from 1 January 2021 to 31 March 2023 in six Grade-A tertiary hospitals in China (China-Japan Friendship Hospital, Dongzhimen Hospital of Beijing University of Chinese Medicine, Dongfang Hospital of Beijing University of Chinese Medicine, Third Affiliated Hospital of Beijing University of Chinese Medicine, Beijing First Hospital of Integrated Traditional Chinese and Western Medicine, and Fangshan Hospital of Beijing University of Chinese Medicine). The improvement of clinical phenotypes before and after 90 days of WSY treatment was observed, including syndrome score, 24-h urine protein (24h UPro), serum creatinine (Scr), estimated glomerular filtration rate (eGFR), albumin-creatinine ratio (ACR), uric acid (UA), blood urea nitrogen (BUN), hemoglobin (Hb), total protein (TP), albumin (ALB), diastolic blood pressure (DBP), systolic blood pressure (SBP). The study protocol was approved by the Ethics Committee of Beijing University of Chinese Medicine (ethics approval number: 2020BZYLL0514.). The study was performed in accordance with the principles of Good Clinical Practice and the Declaration of Helsinki. The study protocol was registered at the China Clinical Trial Registry (ChiCTR) (https://www.chictr.org.cn): ChiCTR2000039644. All participants provided informed written consent. The data were entered into an online system (http://shen.huashuyiwei.com/#/login) by independent individuals who were blinded to the information.

#### 2.1.2 Inclusion and exclusion criteria

Eligible participants needed to meet the following inclusion criteria: 1) Satisfy the Kidney disease: improving global outcomes (KDIGO) clinical practice Guideline for the Evaluation and management for CKD (2012 edition). 2) Patients who had CKD clinical stage 1–4, with eGFR ≥ 30 mL/(min·1.73 m^2^). 3) Patients who were aged 18–75 years 4) Satisfy the TCM syndrome standard according to the TCM syndrome differentiation. The exclusion criteria were as follows: 1) Acute kidney injury that occurred for nearly a month or history of prior of renal replacement therapy. 2) Complicated by acute infectious diseases, severe cardiovascular diseases, primary diseases such as lung and liver system disease, or mental illness. 3) Women who are currently or expected to be pregnant. and 4) Patients who, at the investigator’s discretion, should not participate in this clinical trial.

### 2.2 Metabolomics analysis

#### 2.2.1 Sample collection and pretreatment

Plasma samples were collected by centrifugation at 110 g for 15 min and the top layer of plasma was stored at −80°C for future use. The samples were analyzed for metabolites by Ultra-high-performance liquid chromatography-tandem mass spectrometry (UPLC-MS/MS). Briefly, 100 µL of the sample was thawed at 4°C, vortexed, and then 400 µL of methanol was added and vortexed again. The mixture was sonicated in an ice bath for 20 min and then allowed to stand at −20°C for 1 h. The mixture was centrifuged at 16,000 g for 20 min at 4°C and the supernatant was removed. The supernatant was dried in a high-speed vacuum centrifuge. For mass spectrometry detection, 10 µL of methanol-water solution (1:1, v/v) was added and vortexed for 1 min, then sonicated in an ice bath for 3 min. The mixture was centrifuged at 20,000 g for 15 min at 4°C, and the supernatant was injected for analysis.

#### 2.2.2 Chromatography and mass spectrometry analysis

The sample was placed in an automated sampler at 4°C throughout the analysis. The sample was analyzed on a Waters UPLC I-class UHPLC system using a Waters ACQUITY UPLC HSS T3 Column (2.1 × 100 mm, 1.7 µm) (Waters, Milford, MA, United States) as the chromatography column. The injection volume was 5 μL, the column temperature was 40°C, and the flow rate was 200 μL/min. The chromatographic mobile phase A was 0.1% formic acid-water solution, and the chromatographic mobile phase B was acetonitrile. The gradient elution was as follows: positive ion (0–2.5 min, 0 B; 2.5–9 min, 0%–30% B; 9–10 min, 30%–100% B; 10–15.4 min, 100% B; 15.4–15.5 min, 100%–0% B; 15.5–18 min, 0% B). Negative ion (0–2 min, 0 B; 2–2.5 min, 0%–20% B; 2.5–3.5 min, 20%–40% B; 3.5–4.5 min, 40%–50% B; 4.5–10 min, 50%–100% B; 10–15.4 min, 100% B; 15.4–15.5 min, 100%–0% B; 15.5–18 min, 0% B).

Mass spectrometry was performed using a Thermo Q Exactive Plus Orbitrap high-resolution mass spectrometry detector, scanned in positive and negative ion modes, with a scan range of m/z 25-1200. The electrospray ionization source voltage was 3.2 kV, the ion transfer tube temperature was 320°C, the auxiliary gas heater temperature was 350°C, the sheath gas flow rate was 40 L·min^−1^, and the auxiliary gas flow rate was 15 L·min^−1^. The collision energy to initiate MS2 scans was set to a stepped fragmentation voltage of 25, 7, and 40 V.

#### 2.2.3 Data preprocessing

The raw data were aligned, retention time corrected, and peak areas extracted using the MS-DIAL software. Metabolite structure identification was performed using precise mass matching (mass tolerance <10 ppm) and secondary mass spectra matching (mass tolerance <0.01 Da), searching public databases such as HMDB, MassBank, GNPS, and the BP-DB metabolite standard library. For the extracted data, ion peaks with > 50% missing values within groups were deleted from further statistical analysis, and pattern recognition was performed using Python software. Statistical analysis was performed using the Metabo Analyst (http://www.Metaboanalyst.ca) platform to identify group differences through OPLS-DA. Clustering analysis and Kyoto Encyclopedia of Genes and Genomes (KEGG) enrichment analysis were used to understand the high-level functions and applications of cells, organisms, and ecosystems at the molecular level. Spearman’s correlation coefficient was used to analyze the correlation between differential metabolites and clinical phenotypes.

### 2.3 Network pharmacology analysis

#### 2.3.1 Identification of active components and targets of WSY

Using the TCMSP database platform (http://tcmspw.com/tcmsp.php), search for *C. deserticola* (Ma.), *E. sagittatum* (Maxim.), and *D. roosii* (Nakaike.) were searched separately. The selection criteria were based oral bioavailability (OB) ≥ 30% and drug likeness (DL) ≥ 0.18 selection criteria to obtain effective active ingredients and potential targets. They were then uploaded to the UniProt database (https://www.uniprot.org) for standardized mapping of gene names, and were supplemented with literature. At the same time, combined with the domestic and foreign literature and the previous identification results of WSY compound by LC-MS/MS analysis, the active ingredients and potential targets of WSY compound were supplemented.

#### 2.3.2 Prediction of disease targets and syndrome targets

Use the keyword “chronic kidney disease” to search for targets in the Drug Bank (https://www.drugbank.com/), OMIM (https://omim.org/), TTD (https://db.idrblab.net/ttd/), and GeneCards (https://genecards.org/) databases. According to the Guidelines for Clinical Research of New Chinese Medicine Drugs issued by the Ministry of Health, input the syndrome of kidney Yang deficiency into the GeneCards database. such as chills and cold limbs, cold and painful waist and knees, edema, frequent urination at night, pale complexion, soreness and weakness in the waist and knees, clear and long urine, loose stools, mental fatigue, decreased libido, pale tongue or white tongue coating, sunken and thin pulse or sunken and slow pulse. Finally, merge and deduplicate the targets of CKD and kidney Yang deficiency syndrome, which are the targets related to CKD kidney Yang deficiency syndrome.

### 2.4 Metabolomics and network pharmacology combined analysis

The differential metabolites were input into the PubChem (https://pubchem.ncbi.nlm.nih.gov) website, 2D chemical structures were downloaded and uploaded to Swiss target website to screen differential metabolite targets with a Probability* >0.3. Then we identified the intersection targets by Venn online platform (https://bioinfogp.cnb.csic.es/tools/venny/) of WSY, CKD kidney Yang deficiency syndrome target, and metabolite, which were the main targets that WSY regulated the above-mentioned differential metabolites treating CKD kidney Yang deficiency syndrome. Furthermore, we constructed a “Drug-component-differential metabolite” integration network using Cytoscape 3.10 software to locate the metabolic pathways of the intersection targets and predict the core components. Finally, we entered the intersection targets into the STRING database (https://cn.string-db.org/) to construct a protein-protein interaction (PPI) network, which was screened for hub genes.

### 2.5 Molecular docking

Molecular docking is an important technology used to predict the biological activity between drug molecules and proteins. In addition, the core components of WSY and the hub genes were subjected to molecular docking to reflect the binding strength. The crystal structure of the hub genes was obtained from the RCSB PDB database (PDB, https://www.rcsb.org/), and the SDF file of the core compound structure was obtained from PubChem. The preprocessing was carried out using PyMOL and AutoDockTools 1.5.7 software (developed by Scripps Company), and the Vina 1.1.2 program was used to calculate the molecular docking binding energy. When the binding energy is less than −5 kcal/mol, it indicates that the components were well bound to the hub genes.

### 2.6 *In vitro* cellular experiments

#### 2.6.1 Materials

Human Kidney 2 cells (HK-2) were obtained from ATCC, CRL-2190. Doxorubicin hydrochloride (ACMEC-Biochemical, D91930). DMSO (Solarbio, D8371). Quercetin (Macklin, Q817162). Luteolin (Macklin, L812409). Kaempferol (Macklin, K812225). DMEM/F12 medium (Invitrogen, C11330500BT). Fetal Bovine Serum (Servicebio, G8002). 0.25% Trypsin Digestion solutions (Servicebio, G4001). CCK-8 kit (Biorigin, BN15201). TriZol (LABLEAD, R1000). cDNA reverse transcription kit (Promega, A3500). Eastep^®^ qPCR Master Mix (Promega, LS 2062).

#### 2.6.2 Methods

##### 2.6.2.1 Cell culture

HK-2 cells were cultured in DMEM/F12 medium supplemented with 5% FBS and 1% penicillin-streptomycin, and incubated at 37°C in a 5% CO_2_ incubator.

##### 2.6.2.2 Effect of WSY core components on DOX-induced HK-2 cell viability assessed by CCK-8 assay

Furthermore, we used the CCK-8 assay kit to investigate the effect of different doses of the core component on DOX-induced HK-2 cell viability *in vitro* cell experiments. The peak cell viability was used as the administration dose, which provided the basis for subsequent experiments. HK-2 cells were seeded at a density of 5000 cells per well in 96-well plates and cultured in an incubator at 37°C with 5% CO2. After 24 h, the cells were then divided into the following groups: control group (5% FBS DMEM/F12), DOX group (5% FBS DMEM/F12 + 1 μg/mL DOX) (Li et al., 2019), three core active components (5 μM, 10 μM, 20 μM, 40 μM, 80 μM), and baseline group. The control group was added with 5% FBS DMEM/F12 medium, the DOX group and each core component group were added with 1 μg/mL DOX dilution (diluted with 5% FBS DMEM/F12 medium), and then the medium or drug was added. After 48 h of incubation, the 96-well culture plate was removed. 10 μL CCK-8 and 100 μL DMEM/F12 medium mixture were added to each well, and the culture was continued for 1 h. The absorbance *A* value of each well was detected at the 450 nm wavelength of the microplate reader, and the cell viability was calculated.

##### 2.6.2.3 Effect of the co-administration of core components on DOX-induced HK-2 cell viability assessed by CCK-8 assay

TCM has the characteristics of multi-component and multi-target. After determining the optimal concentration of three core active components, we aimed to further explore whether the co-administration of all three has interaction and synergistic effect on DOX-induced HK-2 cells. In this study, three dosage combinations were set up, and the optimal concentrations of the three core active components were combined in an equal ratio of 1:1:1. After 48 h of culture, CCK8 assay was used to determine the viability of HK-2 cells induced by DOX. The cells were grouped as follows: control group, DOX group (5%FBS DMEM/F12 + 1 ug/mL DOX), Comb 1 group (40 μM is the optimal dose of quercetin, so in Comb 1 group, the quercetin is 40/3 μM, luteolin 40/3 μM, kaempferol 40/3 μM), Comb 2 group (20 μM is the optimal dose of luteolin, so in Comb 2 group, the luteolin 20/3 μM, quercetin is 20/3 μM, kaempferol 20/3 μM), Comb 3 group (10 μM is the optimal dose of kaempferol, so in Comb 3 group, the kaempferol is 10/3 μM, luteolin 10/3 μM, quercetin 10/3 μM). The other experimental procedures were the same as in section 2.6.2.2.

##### 2.6.2.4 RT-qPCR was used to detect the mRNA expression levels of hub genes and related targets

After HK-2 cells were cultured for 48 h, 1 mL of precooled TriZol lysate was added, homogenized, and left on ice for 10 min. Chloroform was added and shaken for 15 s. The mixture was left on ice for 5 min. The mixture was centrifuged at 13,400 g for 15 min at 4°C, and an equal volume of isopropanol was added to the upper layer. The mixture was mixed and kept on ice for 10 min. After centrifugation again under the same conditions, white RNA precipitation was observed. The supernatant was discarded and the precipitate was washed with 75% ethanol. The cells were centrifuged again for 5 min. The samples were dried and precipitated for 10 min at room temperature. RNA concentration and purity were measured by adding 40 μL of ribonuclease-free water to completely dissolve the RNA. Primer sequences are listed in Supplementary Table S1.

### 2.7 Statistical analysis

The experimental data was expressed in the form of mean ± standard deviation. *Paired t*-test was used to analyze the differences of clinical parameters before and after treatment. *One-way ANOVA* was used for variance analysis, and *p* < 0.05 was considered to indicate statistically significant differences.

## 3 Results

### 3.1 Clinical efficacy analysis of WSY

After WSY treatment, the symptoms of kidney Yang deficiency of the patients were significantly improved, 24h UPro, Scr, ACR, DBP were lower than before treatment, eGFR was higher than before treatment, the difference was statistically significant (*p* < 0.05), the results indicated that the clinical efficacy of WSY was significant (Supplementary Table S2).

### 3.2 Analysis of metabolomics results

#### 3.2.1 The metabolic network changed after WSY intervention

**FIGURE 1:**
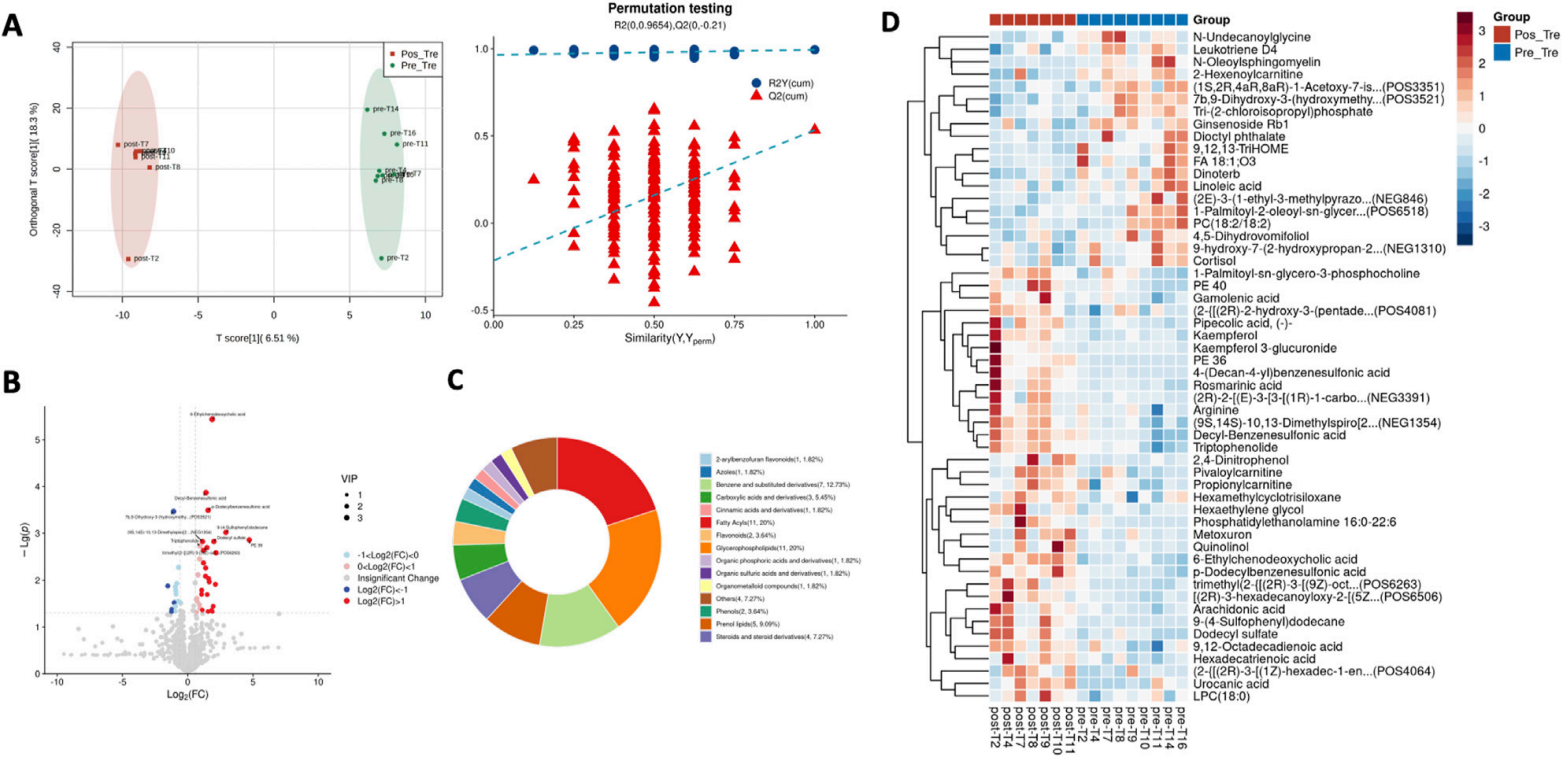
Metabolic Profile Analysis of Differential Metabolites. **(A)**: OPLS-DA showing metabolic profiles for two groups of data. **(B)**: Differential metabolite volcano plot, red dots represent upregulation, blue dots represent downregulation, and dot size represents VIP value magnitude. **(C)**: Ontology classification of differential metabolites. **(D)**: Clustering analysis of differential metabolites.

**FIGURE 2:**
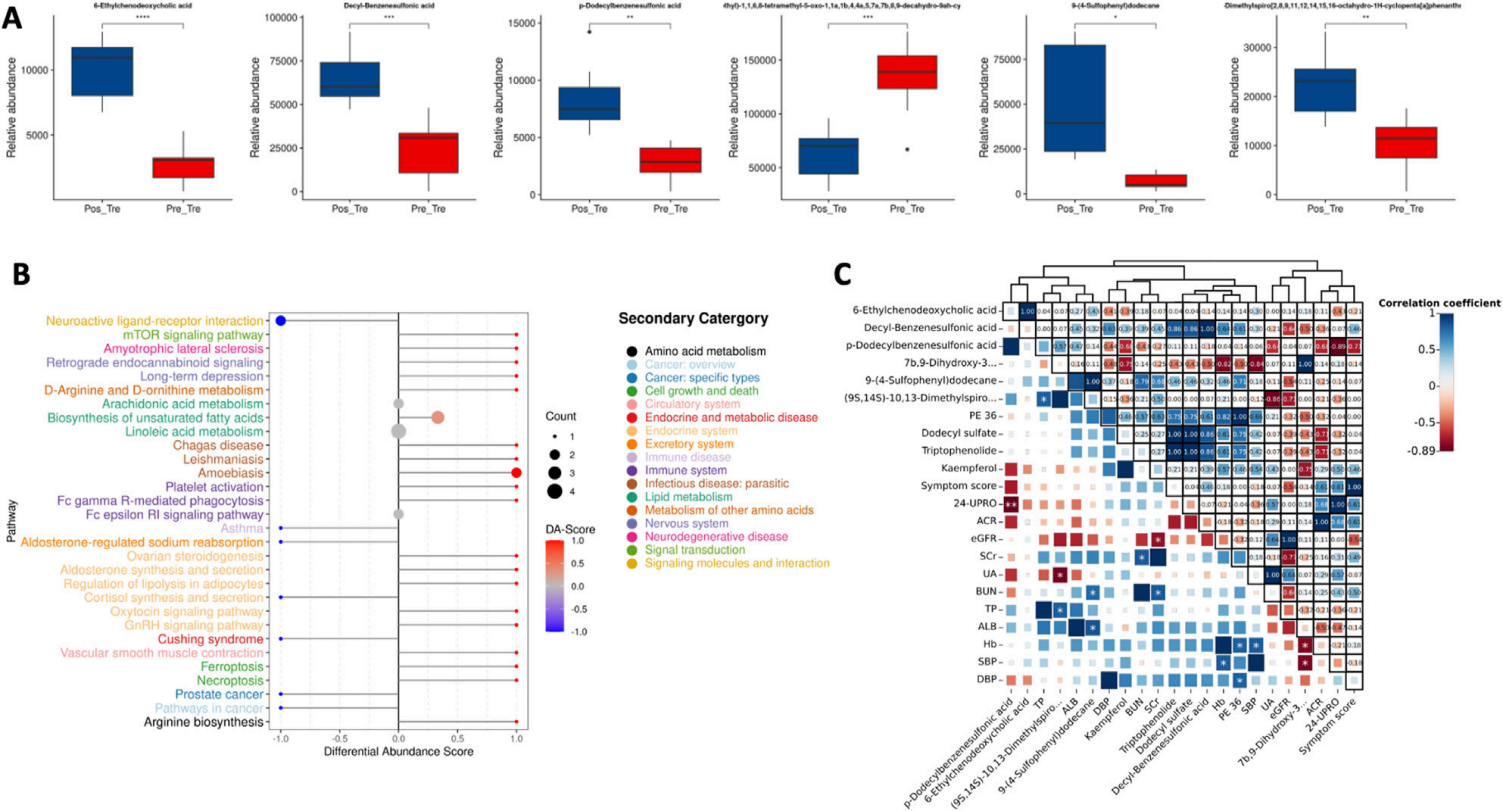
Differential metabolite profiling analysis and correlation analysis. **(A)**: Abundance analysis of major differential metabolites. **(B)**: KEGG enrichment analysis. **(C)**: Correlation analysis between differential metabolites and clinical phenotypes.

In Figure 1A, OPLS-DA shows the metabolic profile of two data sets, with R^2^X = 0.338, R^2^Y = 0.995, Q^2^ = 0.534, and 200 permutation tests show that the intercept of the Q^2^ vertical axis is less than 0, indicating significant differences between the treatment and control groups, and the model has good discriminatory, adaptive and predictive ability. According to the results of OPLS-DA, 54 differential metabolites were identified by screening with VIP >1 and *p* < 0.05 (Table 1; Figures 1B, C). Among them, the concentrations of 35 metabolites increased, including 6-Ethylchenodeoxycholic acid, Decyl-Benzenesulfonic acid, p-Dodecylbenzenesulfonic acid, 9-(4-Sulfophenyl)dodecane, (9S,14S)-10,13, while the relative levels of 19 metabolites decreased, such as 7b,9-Dihydroxy-3-(hydroxymethyl), acetate, N-Oleoylsphingomyelin, 9,12,13-TriHOME, (1S,2R,4aR,8aR)-1-Acetoxy-7. Therefore, we speculated that WSY may achieve the therapeutic effect of treating CKD kidney Yang deficiency syndrome by regulating these metabolites (Figures 1D, 2A).

**TABLE 1:** Differential metabolite.

Alignment ID	Metabolite name	Adduct type	Formular	*P*.value	Log2FC	OPLSDA.VIP
Upregulation						
NEG1900	6-Ethylchenodeoxycholic acid	[M-H]-	C26H44O4	3.66E-06	1.87E+00	3.43E+00
NEG1108	Decyl-Benzenesulfonic acid	[M-H]-	C16H26O3S	1.36E-04	1.40E+00	3.19E+00
NEG1254	p-Dodecylbenzenesulfonic acid	[M-H]-	C18H30O3S	3.22E-04	1.56E+00	3.06E+00
NEG1256	9-(4-Sulfophenyl)dodecane	[M-H]-	C18H30O3S	9.57E-04	2.92E+00	2.91E+00
NEG3422	PE 36	[M-H]-	C41H72NO8P	1.39E-03	4.71E+00	2.88E+00
NEG905	Dodecyl sulfate	[M-H]-	C12H26O4S	1.50E-03	2.00E+00	2.85E+00
NEG1354	(9S,14S)-10,13-Dimethylspiro [2,8,9,11,12,14,15,16-octahydro-1H-cyclopenta [a]phenanthrene-17,5′-oxolane]-2′,3-dione	[M-H]-	C22H28O3	1.50E-03	1.13E+00	2.90E+00
NEG1178	Triptophenolide	[M-H]-	C20H24O3	1.86E-03	9.26E-01	2.83E+00
POS6263	trimethyl (2-{[(2R)-3-[(9Z)-octadec-9-enoyloxy]-2-(tetradecanoyloxy)propyl phosphonato]oxy}ethyl)azanium	[M + H]+	C40H78NO8P	2.02E-03	1.47E+00	2.74E+00
NEG1023	Kaempferol	[M-H]-	C15H10O6	2.31E-03	1.20E+00	2.80E+00
NEG1109	4-(Decan-4-yl)benzenesulfonic acid	[M-H]-	C16H26O3S	2.61E-03	2.17E+00	2.76E+00
POS4255	1-Palmitoyl-sn-glycero-3-phosphocholine	[M + H]+	C24H50NO7P	3.54E-03	9.08E-01	2.63E+00
POS479	Urocanic acid	[M + H]+	C6H6N2O2	4.30E-03	1.21E+00	2.62E+00
NEG1135	Arachidonic acid	[M-H]-	C20H32O2	5.52E-03	1.37E+00	2.64E+00
NEG986	9,12-Octadecadienoic acid	[M-H]-	C18H32O2	7.72E-03	7.86E-01	2.58E+00
NEG1509	Rosmarinic acid	[M-H]-	C18H16O8	8.25E-03	1.36E+00	2.46E+00
POS6506	[(2R)-3-hexadecanoyloxy-2-[(5Z,8Z,11Z,14Z,17Z)-icosa-5,8,11,14,17-pentaenoyl]oxypropyl] 2-(trimethylazaniumyl)ethyl phosphate	[M + H]+	C44H78NO8P	9.23E-03	1.60E+00	2.44E+00
POS403	Pipecolic acid, (−)-	[M + H]+	C6H11NO2	1.08E-02	1.65E+00	2.46E+00
POS2095	Hexaethylene glycol	[M + H]+	C12H26O7	1.14E-02	5.70E-01	2.53E+00
NEG3448	Phosphatidylethanolamine 16:0-22:6	[M-H]-	C43H74NO8P	1.22E-02	2.12E+00	2.45E+00
POS1682	Pivaloylcarnitine	[M + H]+	C12H23NO4	1.63E-02	1.07E+00	2.27E+00
NEG817	Hexadecatrienoic acid	[M-H]-	C16H26O2	2.00E-02	1.06E+00	2.26E+00
NEG3477	PE 40	[M-H]-	C45H76NO8P	2.02E-02	1.49E+00	2.16E+00
NEG404	2,4-Dinitrophenol	[M-H]-	C6H4N2O5	2.44E-02	7.11E-01	2.06E+00
NEG350	Arginine	[M-H]-	C6H14N4O2	2.56E-02	6.50E-01	2.26E+00
POS4541	LPC(18:0)	[M + H]+	C26H50NO7P	3.31E-02	8.97E-01	2.09E+00
POS1385	Propionylcarnitine	[M + H]+	C10H19NO4	3.54E-02	4.61E-01	2.04E+00
POS1502	Metoxuron	[M + H]+	C10H13ClN2O2	3.60E-02	1.95E+00	2.14E+00
NEG976	Gamolenic acid	[M-H]-	C18H30O2	3.95E-02	9.31E-01	2.06E+00
NEG3391	(2R)-2-[(E)-3-[3-[(1R)-1-carboxy-2-(3,4-dihydroxyphenyl)ethoxy]carbonyl-2-(3,4-dihydroxyphenyl)-7-hydroxy-2,3-dihydro-1-benzofuran-4-yl]prop-2-enoyl]oxy-3-(3,4-dihydroxyphenyl)propanoic acid	[M-H]-	C36H30O16	4.33E-02	1.12E+00	1.97E+00
POS564	Quinolinol	[M + H]+	C9H7NO	4.36E-02	1.84E+00	2.03E+00
POS1431	Hexamethylcyclotrisiloxane	[M + H]+	C6H18O3Si3	4.45E-02	3.98E-01	2.01E+00
NEG2190	Kaempferol 3-glucuronide	[M-H]-	C21H18O12	4.66E-02	1.88E+00	2.00E+00
POS4064	(2-{[(2R)-3-[(1Z)-hexadec-1-en-1-yloxy]-2-hydroxypropyl phosphonato]oxy}ethyl)trimethylazanium	[M + H]+	C24H50NO6P	4.68E-02	1.55E+00	1.99E+00
POS4081	(2-{[(2R)-2-hydroxy-3-(pentadecanoyloxy)propyl phosphonato]oxy}ethyl)trimethylazanium	[M + H]+	C23H48NO7P	4.82E-02	6.84E-01	1.99E+00
Down regulation						
POS3521	7b,9-Dihydroxy-3-(hydroxymethyl)-1,1,6,8-tetramethyl-5-oxo-1,1a,1b,4,4a,5,7a,7b,8,9-decahydro-9ah-cyclopropa [3,4]benzo [1,2-e]azulen-9a-yl acetate	[M + ACN + H]+	C22H30O6	3.42E-04	−1.08E+00	3.04E+00
NEG737	Dinoterb	[M-H]-	C10H12N2O5	5.36E-03	−7.01E-01	2.56E+00
POS6244	N-Oleoylsphingomyelin	[M + H]+	C41H81N2O6P	1.32E-02	−1.53E+00	2.38E+00
NEG1289	9,12,13-TriHOME	[M-H]-	C18H34O5	1.38E-02	−9.52E-01	2.28E+00
POS1786	2-Hexenoylcarnitine	[M + NH4]+	C13H23NO4	1.65E-02	−9.11E-01	2.23E+00
POS3351	(1S,2R,4aR,8aR)-1-Acetoxy-7-isopropylidene-1,4a-dimethyl-6-oxodecahydro-2-naphthalenyl 2,3-dimethyl-2-oxiranecarboxylate	[M + Na]+	C22H32O6	1.97E-02	−8.94E-01	2.24E+00
POS7229	Ginsenoside Rb1	[M + H]+	C54H92O23	2.81E-02	−7.22E-01	2.14E+00
NEG1290	FA 18:1; O3	[M-H]-	C18H34O5	2.89E-02	−9.70E-01	2.10E+00
NEG2439	Leukotriene D4	[M-H]-	C25H40N2O6S	2.97E-02	−5.86E-01	2.20E+00
POS6518	1-Palmitoyl-2-oleoyl-sn-glycero-3-phosphocholine	[M + Na]+	C42H82NO8P	3.02E-02	−1.04E+00	2.13E+00
POS2531	Tri-(2-chloroisopropyl)phosphate	[M + H]+	C9H18Cl3O4P	3.04E-02	−5.80E-01	2.09E+00
NEG1310	9-hydroxy-7-(2-hydroxypropan-2-yl)-1,4a-dimethyl-2,3,4,9,10,10a-hexahydrophenanthrene-1-carboxylic acid	[M-H]-	C20H28O4	3.08E-02	−5.35E-01	2.13E+00
NEG846	(2E)-3-(1-ethyl-3-methylpyrazol-4-yl)-1-(4-hydroxyphenyl)prop-2-en-1-one	[M-H]-	C15H16N2O2	3.60E-02	−9.37E-01	2.14E+00
POS3141	Dioctyl phthalate	[M + H]+	C24H38O4	4.18E-02	−1.23E+00	2.00E+00
NEG757	N-Undecanoylglycine	[M-H]-	C13H25NO3	4.49E-02	−5.51E-01	2.02E+00
NEG1817	Cortisol	[M + FA-H]-	C21H30O5	4.56E-02	−5.60E-01	1.99E+00
NEG987	Linoleic acid	[M-H]-	C18H32O2	4.60E-02	−9.66E-01	1.89E+00
POS6520	PC(18:2/18:2)	[M + H]+	C44H80NO8P	4.71E-02	−1.25E+00	1.99E+00
NEG671	4,5-Dihydrovomifoliol	[M-H]-	C13H22O3	4.76E-02	−3.65E-01	2.01E+00

#### 3.2.2 WSY could improve the metabolic pathway disorder

To provide a more comprehensive and intuitive display of the expression pattern differences of metabolites, further ontology metabolite classification and metabolic pathway enrichment analysis was conducted on the 54 biomarkers. The results indicated that the 54 differential metabolites were associated with phosphatidylcholines, fatty acyls, glycerophospholipids, benzene and substituted derivatives, prenol lipids, steroids and steroid derivatives. The KEGG enrichment analysis revealed that WSY exerts a regulatory influence on a number of metabolic processes, including those related to linoleic acid metabolism, the FcεRI signaling pathway, the biosynthesis of unsaturated fatty acids, neuroactive ligand-receptor interaction, the mTOR signaling pathway, and arachidonic acid metabolism (Figure 2B).

#### 3.2.3 Differential metabolites were correlated with clinical phenotypes

In Figure 2C, the spearman correlation coefficients were calculated between differential metabolites and clinical phenotypes. The results showed that sodium p-Dodecylbenzenesulfonic acid was negatively correlated with 24h UPro. (9S,14S)-10,13-Dimethylspiro. was negatively correlated with UA, and positively correlated with TP, and 9-(4-sulfophenyl)dodecane was positively correlated with BUN and ALB. 7b,9-Dihydroxy-3-...was negatively correlated with Hb and SBP. PE 36 was positively correlated with Hb and DBP. Therefore, it can be postulated that the enhancement of renal function indices is associated with the change of these metabolite concentrations.

### 3.3 WSY, CKD, syndrome factor targets were obtained by network pharmacology

Combined with the results of the TCMSP platform and the results of the LC-MS/MS analysis, a total of 59 chemical components of WSY were identified. After removing the chemical components that were not predicted by the TCMSP platform and the Swiss target database, 46 effective active components of WSY remained. After UniProt database unification of target names and removal of duplicate values, 252 potential targets of WSY were obtained (Supplementary Tables S3, S4; Supplementary Figure S1).

A total of 956 targets for CKD and 1742 targets for kidney Yang deficiency syndrome were identified. There were 286 targets of “loose stool” syndrome, 228 targets of “mental fatigue” syndrome, 341 targets of “sunken and thin pulse or sunken and slow pulse” syndrome, 291 targets of “pale complexion” syndrome, 39 targets of “white tongue coating” syndrome, 266 targets of “edema” syndrome, 194 targets of “chills and cold limb” syndrome, 1014 targets of “clear and long urine” syndrome, 74 targets of “decreased libido” syndrome, 102 targets of “cold and painful waist and knees” syndrome, 114 targets of “soreness and weakness in the waist and knees” syndrome, and 366 targets of “frequent urination at night” syndrome.

### 3.4 Metabolomics and network pharmacology combined analysis

#### 3.4.1 Quercetin, luteolin and kaempferol were predicted to be the core components of WSY

**FIGURE 3:**
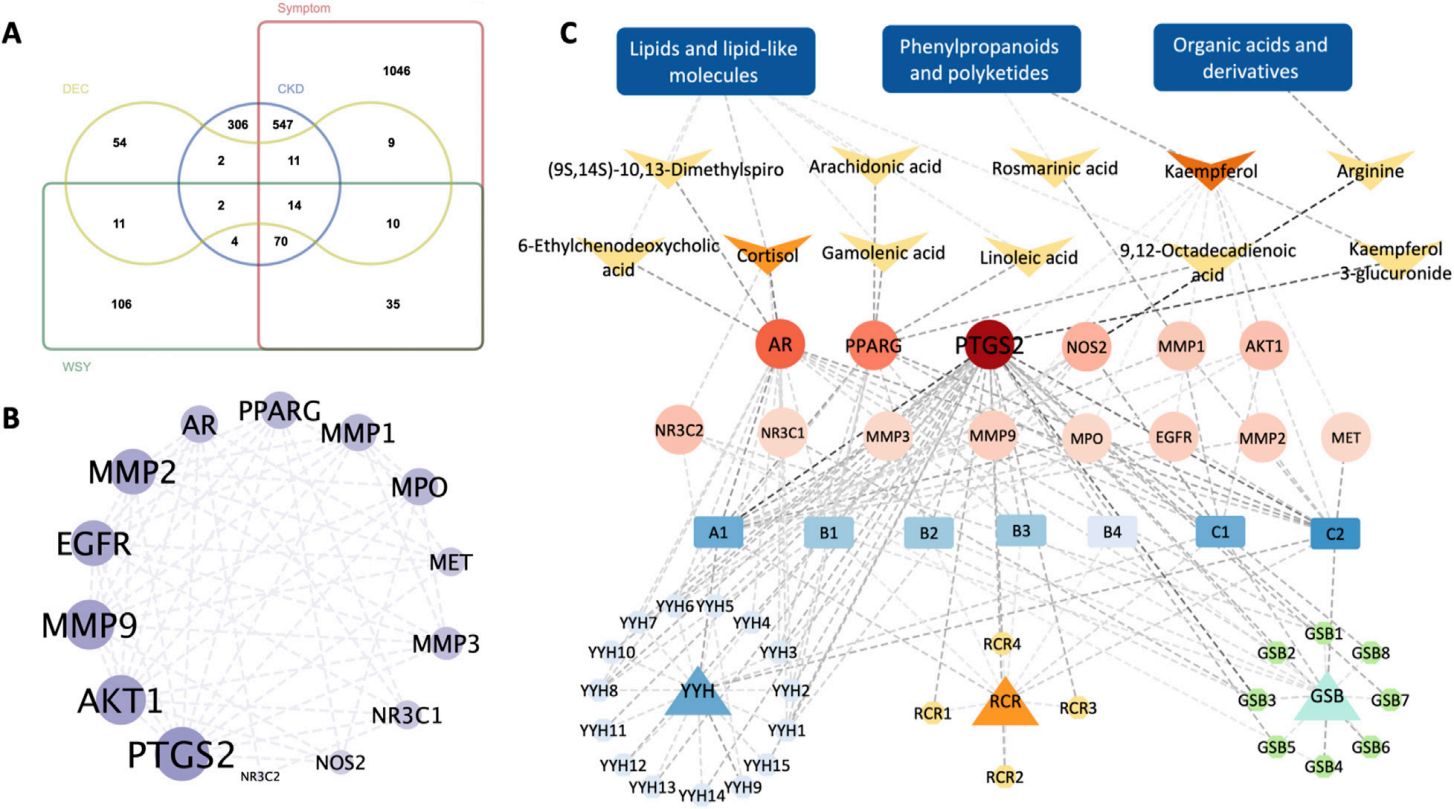
Integrative network pharmacology analysis of differential metabolites. **(A)**: Venn diagram of differential metabolites, WSY drug components, diseases, and syndrome targets. **(B)**: Key targets of PPI. **(C)**: Integrated network of “Drug-Component-Differential Metabolite” [Note: RCR-*Cistanche deserticola* (Ma.), YYH-*Epimedium sagittatum* (Maxim.), GSB-*Drynaria roosii* (Nakaike.)].

Furthermore, we matched the differential metabolites with the substances and molecules in the metabolite databases HMDB and KEGG and obtained 560 targets corresponding to the differential metabolites. We combined the differential metabolite targets, WSY, disease and syndrome factor targets, and finally obtained 14 intersection targets. These targets were the key targets of WSY regulating the above differential metabolites in the treatment of CKD kidney Yang deficiency syndrome (Figure 3A).

On this basis, we constructed the “Drug-Component-Differential Metabolite” intersection network and reverse-predicted the core components of WSY, which indicates that these 14 key targets are involved in three metabolic pathways, such as lipids and lipid-like molecules, phenylpropanoids and polyketides, and organic acids and derivatives, and 11 differential metabolites, including (9S,14S)-10,13-Dimethylspiro, cortisol, 6-Ethylchenodeoxycholic acid, 9,12-Octadecadienoic acid, arachidonic acid, linoleic acid, gamolenic acid, kaempferol 3-glucuronide, kaempferol, rosmarinic acid, arginine, and 34 WSY core components. Based on the degree value of the network, we predicted that quercetin, luteolin and kaempferol might be the main material basis for the therapeutic effect of WSY.

#### 3.4.2 PTGS2, AKT1, MMP9, MMP2, and EGFR were predicted to be the hub genes

In Figure 3B, the 14 key targets were imported into the STRING database to construct the PPI network. Based on the degree values, we hypothesized that PTGS2, AKT1, MMP9, MMP2, and EGFR could be the hub genes. Combined with the “Drug-Component-Differential Metabolite” integrated network, we localized the key targets, and the results showed that AR, NR3C2, NR3C1, PPARG are involved in the biosynthesis of lipids and lipid-like molecules. PTGS2, MMP3, EGFR, AKT1, MMP2, MMP9, MPO, MET, MMP1 in the biosynthesis of phenylpropanoids and polyketides. NOS2 in the biosynthesis of organic acids and derivatives (Figure 3C).

### 3.5 Molecular docking showed that the core components strongly bind to the hub genes

**FIGURE 4:**
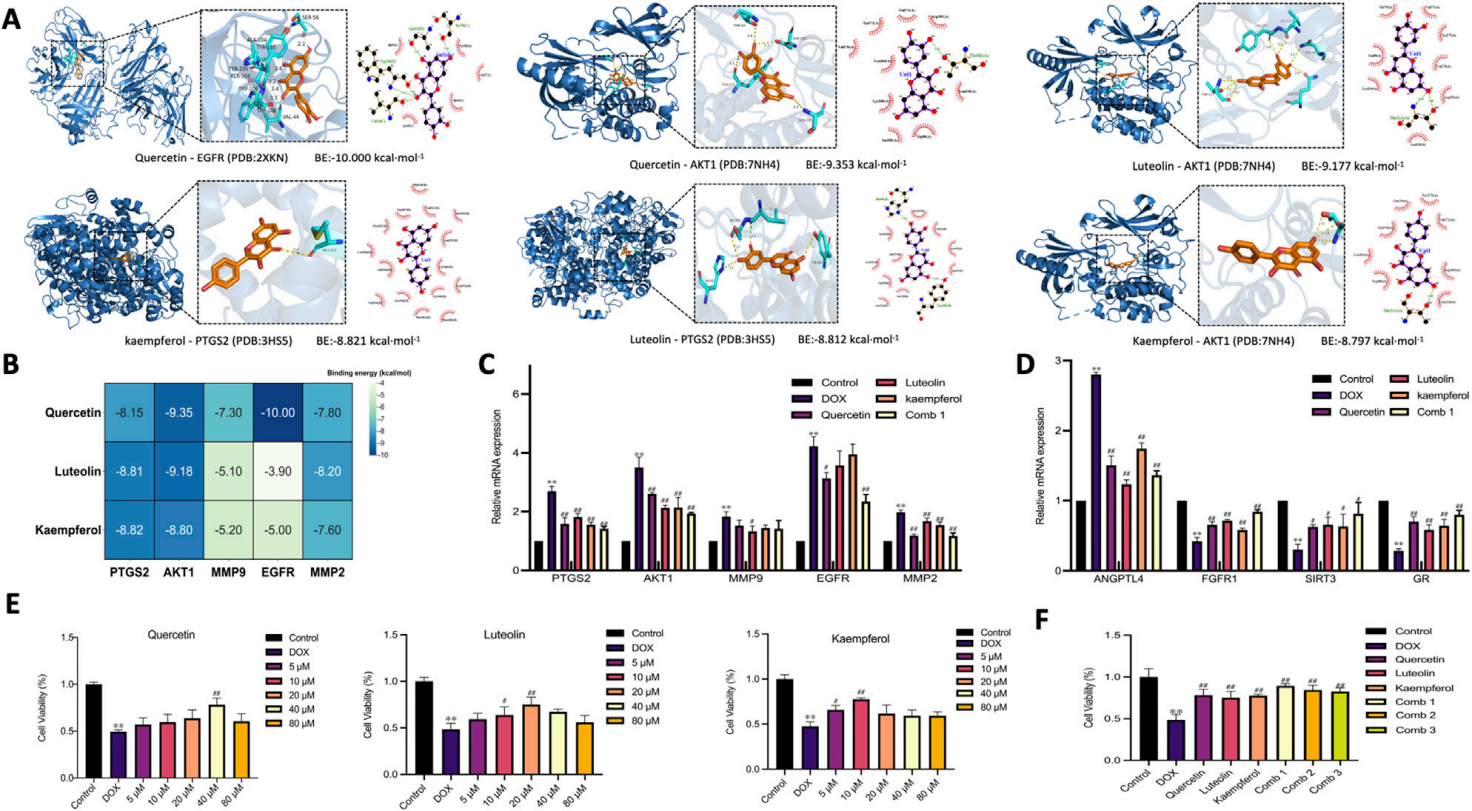
Molecular docking of WSY core components and core target molecules and experimental verification. **(A)**: The strongest binding capacity of the top 6; **(B)**: The results of molecular docking of the three core component with five hub genes. **(C, D)**: The mRNA levels of hub genes and related targets were determined by RT-qPCR. **(E)**: Effect of WSY core component on DOX-induced HK-2 cell viability assessed by CCK-8 assay. **(F)**: Effect of the co-administration of core component on DOX-induced HK-2 cell viability assessed by CCK-8 assay. Annotation: Compared with conteol group,^*^ *p* < 0.05, ^**^ *p* < 0.01. Compared with DOX group, ^#^ *p* < 0.05, ^##^ *p* < 0.01.

We performed molecular docking of the three core active components with five hub genes (Figure 4B). The AutoDock calculation results showed the strongest binding capacity of the top 6: Quercetin - EGFR (PDB:2XKN) was −10.000 kcal/mol, the binding energy of Quercetin - AKT1 (PDB:7NH4) was - 9.353 kcal/mol, the binding energy of Luteolin - AKT1 (PDB:7NH4) was −9.177 kcal/mol, the binding energy of kaempferol - PTGS2 (PDB:3HS5) was −8.821 kcal/mol, the binding energy of Luteolin - PTGS2 (PDB:3HS5) was −8.812 kcal/mol, and the binding energy of Kaempferol - AKT1 (PDB:7NH4) was −8.797 kcal/mol (Figure 4A). These results indicated that quercetin, luteolin and kaempferol were stable in binding to the ligand proteins.

### 3.6 Quercetin, luteolin and kaempferol could increase the viability of HK-2 cells induced by DOX

To further verify the reliability of the prediction results, the CCK-8 assay was used to investigate the effects of different doses of quercetin, luteolin and kaempferol on the viability of HK-2 cells induced by DOX. As shown in Figure 4E, DOX significantly inhibited the viability of HK-2 cells compared with the control group (*p* < 0.05), resulting in the loss of cell viability. Compared with the DOX group, the cell viability of the quercetin group, luteolin group, and kaempferol group was increased to different degrees (*p* < 0.05). Among them, the optimal dose of quercetin was 40 μM, luteolin was 20 μM, and kaempferol was 10 μM. These results indicated that different concentrations of quercetin, luteolin, and kaempferol could upregulate the viability of HK-2 cells and alleviate the cell damage to some extent.

### 3.7 Co-administration of core active components was superior to single active component in inducing DOX-induced HK-2 cell viability

TCM compound is an organic whole composed of multiple herbs and multiple components, and the material basis of its disease treatment is the sum of all chemical components. To further explore whether the co-administration of all three core active component have interaction and synergistic effect on DOX-induced HK-2 cells. We validated the effect of three different dose combinations. As shown in Figure 4F, the results showed that compared with the DOX group, the cell viability of the core active components group was significantly increased (*p* < 0.05), and the combined treatment group had a better effect than the single active group, among which the Comb 1 group had the most significant effect (*p* < 0.05).

### 3.8 Quercetin, kaempferol, and luteolin may play an anti-inflammatory and anti-fibrosis role by regulating the mRNA expression levels of key genes and related targets

After determining the optimal concentrations of quercetin, luteolin, kaempferol and their co-administration, RT-qPCR was used to determine the mRNA expression levels of hub genes (PTGS2, AKT1, MMP9, EGFR and MMP2) and related targets (ANGPTL4, FGFR1, SIRT3, GR) in HK-2 cells induced by DOX. The results showed that compared with the control group, the mRNA levels of PTGS2, AKT1, MMP9, EGFR, MMP2, and ANGPTL4 were significantly upregulated, and the levels of FGFR1, SIRT3, and GR were significantly downregulated in the DOX group (*p* < 0.05). Compared with the DOX group, PTGS2, AKT1, MMP9, EGFR, MMP2, and ANGPTL4 were decreased in the quercetin group, luteolin group, kaempferol group, and Comb 1 group. FGFR1, SIRT3 and GR were increased to different degrees (*p* < 0.05), and the effect of combined treatment group was more significant (*p* < 0.05) (Figures 4C, D).

## 4 Discussion

We here found that metabolites were significantly changed after WSY treatment. A total of 54 differential metabolites were obtained in POS and NEG modes, mainly involved in the metabolism of glycerophospholipids, fatty acyls and other substances. A study demonstrated that lipids play an important role in regulating various important life activities, including substance transport, energy conversion, cell differentiation and apoptosis. Lipid metabolism disorders are closely related to inflammatory responses and immune responses (Du et al., 2015). Previous studies have shown that CKD kidney Yang deficiency syndrome patients have disorders in glycerophospholipid metabolism, and arachidonic acid metabolism (Fu et al., 2019). KEGG enrichment analysis showed that WSY mainly disrupted linoleic acid metabolism, FcεRI signaling pathway, biosynthesis of unsaturated fatty acids, arachidonic acid metabolism. Linoleic acid is an important component of enzymes and has functions such as inhibiting inflammation and lowering blood lipid levels. It belongs to polyunsaturated fatty acids (PUFAs). Research have shown that consuming foods rich in linoleic acid can lower plasma total cholesterol, lipoprotein levels, and reduce urine protein excretion rate and the incidence of glomerular sclerosis (Gaullier et al., 2005; Dos Santos et al., 2018). Another study pointed out that ω-3 PUFAs, as a nutritional treatment method, can improve the inflammatory state of CKD patients to some extent (Zhou et al., 2015). Arachidonic acid is a precursor of important signal transduction molecules such as prostaglandins and leukotrienes, and belongs to unsaturated fatty acids. Studies have shown that the decrease of unsaturated fatty acids is related to the disorder of lipid metabolism, decreased immunity and memory loss in patients with Yang deficiency (Li et al., 2011). Importantly, we demonstrated that the disorder of plasma metabolites was closely related to the changes in renal function indicators, implying that WSY may exert therapeutic effects by disturbing these metabolites.

In addition, we constructed a “Drug-Component-Differential Metabolite” integrated network for further exploration. We predicted that quercetin, luteolin and kaempferol could be the main material basis and AKT1, PTGS2, MMP9, EGFR and MMP2 were the hub genes. Molecular docking results showed that they had good binding capacity with AKT1, PTGS2, MMP9, MMP2 and EGFR. CCK-8 results showed that quercetin, luteolin and kaempferol could significantly increase the viability of HK-2 cells induced by DOX, and the co-administration of all three core active components was better than that of the single group. Quercetin, luteolin and kaempferol are all flavonoid compounds, evidence-based pharmacological data have shown that flavonoids are a class of active natural products with a wide range of pharmacological effects. They have significant anti-inflammatory, antioxidant, anti-stress and anti-fibrosis effects, which play a key role in the prevention and treatment of CKD. Studies have suggested that quercetin can prevent glomerular and tubular damage in diabetic rats by reducing lipid peroxidation and increasing SOD and CAT activities (Elbe et al., 2015). Kaempferol can significantly reduce renal inflammation, fibrosis and renal dysfunction by regulating TRAF6 (Luo et al., 2021). Luteolin may ameliorate glomerulosclerosis and interstitial fibrosis in db/db mouse models by inhibiting the inflammatory response and oxidative stress through suppressing signal transducer and STAT3 activation (Zhang et al., 2021).

PTGS2, AKT1, MMP9, EGFR and MMP2 were the hub genes predicted in the present study, which were closely related to processes of inflammation, endothelial-to-mesenchymal transition (EndMT), fibrosis, oxidative stress, cell migration, proliferation, and apoptosis. Kidney fibrosis is the final consequence of DKD, inflammation has a prominent role in initiating renal fibrosis. PTGS2 is the main isoform of the enzyme that converts arachidonic acid into inflammatory prostaglandins, and is involved in inflammation, the maintenance of sodium and water homeostasis, the regulation of renin release, the dilation of renal blood vessels, the attenuation of vascular constriction, and the development of the kidneys (Rios et al., 2012). Studies have shown that quercetin and kaempferol can affect the metabolism of arachidonic acid by regulating PLA2, COX and LOX, thereby inhibiting the biosynthesis of prostaglandins, thromboxanes and leukotrienes to exert anti-inflammatory effects (Hanáková et al., 2017). Another study showed that quercetin could protect the kidney by inhibiting PTGS2 and TNFα and regulating MAPK and NF-κB inflammatory signalling pathways in a dose-dependent manner (Xiang et al., 2022). AKT1 is a key target of renal fibrosis involved in the transition from acute kidney injury to CKD. Research has indicated that inhibiting AKT phosphorylation can block GSK-3β phosphorylation and restore GSK-3β activity, which contributes to the degradation of β-catenin, thereby preventing EMT. MMP9 and MMP2 belong to the matrix metalloproteinase family and are closely associated with the process of hypertensive nephropathy and renal fibrosis (Yu et al., 2022). MMP-9 inhibition has been shown to decrease neutrophil and other inflammatory cell infiltration and renal fibrosis. (Wang et al., 2019). EGFR is the receptor for epidermal growth factor, which can induce renal tubular epithelial cells to secrete E-cadherin, FSP1, and α-SMA, promote cell migration, and lead to fibrosis. Aberrant EGFR activation is a mediator of progressive kidney injury in diabetic nephropathy (Harris R. C., 2022). In the present study, we demonstrated that that quercetin, luteolin and kaempferol could significantly reduce the mRNA expression levels of PTGS2, AKT1, MMP9, EGFR and MMP2 (*p < 0.05*). Therefore, it is reasonable to speculate that the possible mechanism of WSY in the treatment of CKD is closely related to its core active compounds exert an anti-inflammatory, anti-fiber effects by regulating key targets.

To deepen our understanding, we reviewed the literature and validated the major related targets of anti-inflammatory, anti-EMT and anti-fibrosis treatment of CKD, such as ANGPTL4, FGFR1, SIRT3 and GR. One study suggested that blocking renal ANGPTL4 in podocytes or tubules effectively suppressed proteinuria, glomerular fibrosis, and interstitial fibrosis in diabetic mice (Srivastava et al., 2024). EndMT is one of the key trigger mechanisms for fibrosis, FGFR1 has been identified as a key anti-EndMT molecule (Li et al., 2020). Another study suggested that EndMT alters EC structure and metabolism (Zhu et al., 2023). Interestingly, we have shown through metabolomics analysis that there were marked changes in the metabolic network in the body of patients after WSY intervention, mainly related to energy metabolism and regulation of fatty acid metabolism. SIRT3 is a major mitochondrial deacetylase involved in glycolysis and energy metabolism (Yin and Cadenas, 2015). A study demonstrated that overexpression of SIRT3 in endothelial cells can protect against renal fibrosis by attenuating metabolic reprogramming and the associated mesenchymal transformation (Srivastava et al., 2021a). Endogenous GR act as negative regulators of inflammation (Srivastava and Goodwin, 2023). Previous studies have shown that endothelial GR deficiency results in increased Wnt signaling, aberrant cytokine reprogramming, and suppressed fatty acid oxidation, which can promote injury and fibrotic phenotypes (Srivastava et al., 2021b). Of note, we found that quercetin, luteolin and kaempferol can reduce the levels of ANGPTL4 mRNA and increase the levels of FGFR1, SIRT3 and GR mRNA in HK-2 cells induced by doxorubicin. These results further strengthen our views that the inflammation and fibrosis phenotypes can be partially rescued by the main active ingredients of WSY.

## 5 Conclusion

In conclusion, our findings demonstrated that the mechanism of WSY may be related to the key active ingredients, quercetin, luteolin and kaempferol, by regulating PTGS2, AKT1, MMP9, EGFR, MMP2 and related key targets, and mediating linoleic acid metabolism, FcεRI signalling pathway, unsaturated fatty acid metabolism pathway, and playing a synergistic role in anti-inflammatory and anti-fibrosis. This study also further confirmed that TCM has the characteristics of multi-component, multi-target and multi-pathway in the prevention and treatment of diseases, which provides a methodological reference for the mechanism research in the treatment of CKD.

## Data Availability

The data presented in the study are deposited in the Zenodo repository. Accession: https://zenodo.org/records/14912816.

## Abbreviations

24h UPro, 24-h urine protein; ACR, Albumin-to-creatinine ratio; ALB, Albumin; BUN, Blood urea nitrogen; ChiCTR, China Clinical Trial Registry; Comb, Combination; CKD, Chronic kidney disease; DBP, Diastolic blood pressure; DL, Drug likeness; DOX, Doxorubicin; EndMT, Endothelial-to-mesenchymal transition; eGFR, estimated Glomerular filtration rate; Hb, Hemoglobin; HK-2, Human kidney-2; KDIGO, Kidney disease: improving global outcomes; KEGG, Kyoto Encyclopedia of Genes and Genomes; OB, Oral bioavailability; PPI, Protein-protein interaction; PUFAs, Polyunsaturated fatty acids; RT-qPCR, Reverse transcription quantitative polymerase chain reaction; SBP, Systolic blood pressure; Scr, Serum creatinine; TCM, Traditional Chinese Medicine; TP, Total protein; UA, Uric acid; UPLC-MS/MS, Ultra-high-performance liquid chromatography-tandem mass spectrometry; WSY, Wenshenyang decoction.
