# Supplementary material for: Integrated metabolomics and network pharmacology analysis to reveal the mechanisms of Wenshenyang decoction in the treatment of chronic kidney disease
Source: Front Pharmacol. 2025 Apr 30;16:1500463. doi: 10.3389/fphar.2025.1500463 (PMC12086429; doi:10.3389/fphar.2025.1500463)
Supplement: Supplementary file 1 [file DataSheet1.docx]

Supplementary Material

Contents

[Table S1 Primer sequence information 2](#_Toc441488709)

[Table S2 Baseline data of the patients and primary efficacy analysis 3](#_Toc544731778)

[Table S3 WSY components predicted by network pharmacology 4](#_Toc584205685)

[Table S4 UHPLC Exactive - Orbitrap/MS identification Wenshenyang decoction main chemical composition 6](#_Toc449713711)

[Table S5 Compound component ID 7](#_Toc1343386984)

[Figuer S1 UHPLC Exactive - Orbitrap/MS identification of Wenshenyang decoction 8](#_Toc1809459177)

# Table S1 Primer sequence information

| **Primers** | **Sequence (5'-3')** | **Product length (bp)** |
| --- | --- | --- |
| PTGS2 | F: CCCTTCTGCCTGACACCTTT | 172 |
|  | R: TTCTGTACTGCGGGTGGAAC |  |
| AKT1 | F: TGGACGATAGCTTGGAGGGA | 241 |
|  | R: ATTGTTCTGAGGGCTGAGGC |  |
| MMP9 | F: TTGACAGCGACAAGAAGTGG | 146 |
|  | R: CTCAGTGAAGCGGTACATAGG |  |
| MMP2 | F: CTGGACTTAGACCGCTTGGC | 216 |
|  | R: AAACAGGTTGCAGCTCTCCT |  |
| EGFR | F: AGGCACGAGTAACAAGCTCAC | 177 |
|  | R: ATGAGGACATAACCAGCCACC |  |
| ANGPTL4 | F: CAAGGCTCAGAACAGCAGGA | 298 |
|  | R: CCCCTGAGGCTGGATTTCAA |  |
| FGFR1 | F: gaggacgcaggggagtatac | 93 |
|  | R: ctcttccagggcttccagaa |  |
| SIRT3 | F: ACCCAGTGGCATTCCAGAC | 123 |
|  | R: GGCTTGGGGTTGTGAAAGAAG |  |
| GR | F: AAGAGCAGTGGAAGGACAGC | 128 |
|  | R: CCAGGTTCATTCCAGCCTGA |  |
| β-actin | F: CGGGACCTGACTGACTACC | 292 |
|  | R: TGAAGGTAGTTTCGTGGATGC |  |

# Table S2 Baseline data of the patients and primary efficacy analysis

| **Indicators** | **Before treatment** | **After treatment** | **Difference and 95% confidence interval** | ***t*** | ***p*** |
| --- | --- | --- | --- | --- | --- |
| 24h Upro | 1.73±0.44 | 1.33±0.57 | 0.40(0.14,0.66) | 3.765 | 0.009 |
| Scr | 115.29±11.59 | 94.47±7.89 | 20.82(10.00,31.64) | 4.707 | 0.003 |
| eGFR | 76.40±16.27 | 83.65±17.97 | -7.25(-11.20,-3.29) | -4.483 | 0.004 |
| ACR | 89.14±33.12 | 66.59±21.22 | 22.55(8.30,36.80) | 3.873 | 0.008 |
| UA | 365.14±26.13 | 348.82±31.70 | 16.32(-9.02,41.67) | 1.576 | 0.166 |
| BUN | 8.20±2.87 | 7.81±2.22 | 0.39(-1.15,1.93) | 0.625 | 0.555 |
| TP | 67.51±6.75 | 68.67±7.50 | -1.16(-3.14,0.83) | -1.428 | 0.203 |
| ALB | 40.06±4.02 | 42.35±4.19 | -2.29(-8.51,3.93) | -0.901 | 0.402 |
| Hb | 127.14±13.12 | 137.43±10.05 | -10.29(-20.62,0.05) | -2.435 | 0.051 |
| SBP | 129.43±10.88 | 125.71±9.18 | 3.71（-4.78，12.20） | 1.071 | 0.325 |
| DBP | 80.57±5.62 | 72.57±2.23 | 8（1.96，14.04） | 3.24 | 0.018 |
| Symptom score | 22.14±2.85 | 13.57±4.28 | 8.57(3.95,13.19) | 4.54 | 0.004 |

# Table S3 WSY components predicted by network pharmacology

| **Compound number** | **Component name** | **OB(%)** | **DL** |
| --- | --- | --- | --- |
| MOL000358 | beta-sitosterol | 36.91 | 0.75 |
| MOL005320 | arachidonate | 45.57 | 0.2 |
| MOL005384 | suchilactone | 57.52 | 0.56 |
| MOL007563 | Yangambin | 57.53 | 0.81 |
| MOL000098 | quercetin | 46.43 | 0.28 |
| MOL008871 | Marckine | 37.05 | 0.69 |
| MOL001510 | 24-epicampesterol | 37.58 | 0.71 |
| MOL001645 | Linoleyl acetate | 42.1 | 0.2 |
| MOL001771 | poriferast-5-en-3beta-ol | 36.91 | 0.75 |
| MOL001792 | DFV | 32.76 | 0.18 |
| MOL003044 | Chryseriol | 35.85 | 0.27 |
| MOL003542 | 8-Isopentenyl-kaempferol | 38.04 | 0.39 |
| MOL000359 | sitosterol | 36.91 | 0.75 |
| MOL000422 | kaempferol | 41.88 | 0.24 |
| MOL004367 | olivil | 62.23 | 0.41 |
| MOL004373 | Anhydroicaritin | 45.41 | 0.44 |
| MOL004380 | C-Homoerythrinan, 1,6-didehydro-3,15,16-trimethoxy-, (3.beta.)- | 39.14 | 0.49 |
| MOL004382 | Yinyanghuo A | 56.96 | 0.77 |
| MOL004384 | Yinyanghuo C | 45.67 | 0.5 |
| MOL004386 | Yinyanghuo E | 51.63 | 0.55 |
| MOL004388 | 6-hydroxy-11,12-dimethoxy-2,2-dimethyl-1,8-dioxo-2,3,4,8-tetrahydro-1H-isochromeno[3,4-h]isoquinolin-2-ium | 60.64 | 0.66 |
| MOL004391 | 8-(3-methylbut-2-enyl)-2-phenyl-chromone | 48.54 | 0.25 |
| MOL004394 | Anhydroicaritin-3-O-alpha-L-rhamnoside | 41.58 | 0.61 |
| MOL004396 | 1,2-bis(4-hydroxy-3-methoxyphenyl)propan-1,3-diol | 52.31 | 0.22 |
| MOL004425 | Icariin | 41.58 | 0.61 |
| MOL004427 | Icariside A7 | 31.91 | 0.86 |
| MOL000006 | luteolin | 36.16 | 0.25 |
| MOL000622 | Magnograndiolide | 63.71 | 0.19 |
| MOL001040 | (2R)-5,7-dihydroxy-2-(4-hydroxyphenyl)chroman-4-one | 42.36 | 0.21 |
| MOL001978 | Aureusidin | 53.42 | 0.24 |
| MOL002914 | Eriodyctiol (flavanone) | 41.35 | 0.24 |
| MOL000449 | Stigmasterol | 43.83 | 0.76 |
| MOL004328 | naringenin | 59.29 | 0.21 |
| MOL000492 | (+)-catechin | 54.83 | 0.24 |
| MOL000569 | digallate | 61.85 | 0.26 |
| MOL009061 | 22-Stigmasten-3-one | 39.25 | 0.76 |
| MOL009063 | Cyclolaudenol acetate | 41.66 | 0.79 |
| MOL009075 | cycloartenone | 40.57 | 0.79 |
| MOL009078 | davallioside A_qt | 62.65 | 0.51 |
| MOL009091 | xanthogalenol | 41.08 | 0.32 |

# Table S4 UHPLC Exactive - Orbitrap/MS identification Wenshenyang decoction main chemical composition

| **Number** | **Compound** | **Formula** | ***t*_R_ (min)** | **Top 3 peaks (m/z)** | **Compound classification** |
| --- | --- | --- | --- | --- | --- |
| 1 | HMF/5-hydroxymethylfurfural | C_6_H_6_O_3_ | 0.65 | 109.028, 127.039, 81.033 | Organic matter |
| 2 | Quercetin | C_15_H_10_O_7_ | 1.52 | 303.050, 229.050, 153.018 | Flavonoids |
| 3 | Acteoside | C_29_H_36_O_15_ | 2.16 | 163.039, 325.092, 181.049 | Phenylpropanoid glycosides |
| 4 | Kaempferol | C_15_H_10_O_6_ | 1.58 | 287.055, 153.019, 121.028 | Flavonoids |
| 5 | Eriodictyol | C_15_H_12_O_6_ | 1.70 | 289.071, 153.018, 163.039 | Flavonoids |
| 6 | Betaine | C_5_H_11_NO_2_ | 7.69 | 118.086, 59.060, 87.055 | Alkaloid |
| 7 | Anhydroicaritin | C_21_H_20_O_6_ | 6.08 | 313.071, 369.134, 243.065 | Flavonoids |
| 8 | Isoacteoside | C_29_H_36_O_15_ | 2.16 | 163.039, 325.092, 625.225 | Flavonoids |
| 9 | Icariin | C_33_H_40_O_15_ | 6.08 | 699.225, 311.100, 391.115 | Flavonoids |
| 10 | Cis-resveratrol | C_14_H_12_O_3_ | 9.20 | 369.133, 313.071, 299.055 | Flavonoids |
| 11 | Naringin | C_27_H_32_O_14_ | 2.58 | 273.076, 85.028, 195.029 | Flavonoids |
| 12 | Naringenin | C_15_H_12_O_5_ | 4.05 | 273.076, 153.018, 147.044 | Flavonoids |

# Table S5 Compound component ID

| **Code** | **Compound component ID** | **Code** | **Compound component ID** |
| --- | --- | --- | --- |
| A1 | MOL000098 | YYH11 | MOL001792 |
| B1 | MOL001978 | YYH12 | MOL004367 |
| B2 | MOL004328 | YYH13 | MOL004388 |
| B3 | MOL009091 | YYH14 | MOL004396 |
| B4 | MOL000358 | YYH15 | MOL004427 |
| C1 | MOL000422 | RCR1 | MOL005320 |
| C2 | MOL000006 | RCR2 | MOL005384 |
| YYH1 | MOL003044 | RCR3 | MOL007563 |
| YYH2 | MOL003542 | RCR4 | MOL008871 |
| YYH3 | MOL004373 | GSB1 | MOL000449 |
| YYH4 | MOL004380 | GSB2 | MOL000569 |
| YYH5 | MOL004382 | GSB3 | MOL001040 |
| YYH6 | MOL004384 | GSB4 | MOL009075 |
| YYH7 | MOL004386 | GSB5 | MOL002914 |
| YYH8 | MOL004391 | GSB6 | MOL000492 |
| YYH9 | MOL000359 | GSB7 | MOL005190 |
| YYH10 | MOL001645 | GSB8 | MOL009078 |

# Figuer S1 UHPLC Exactive - Orbitrap/MS identification of Wenshenyang decoction

**
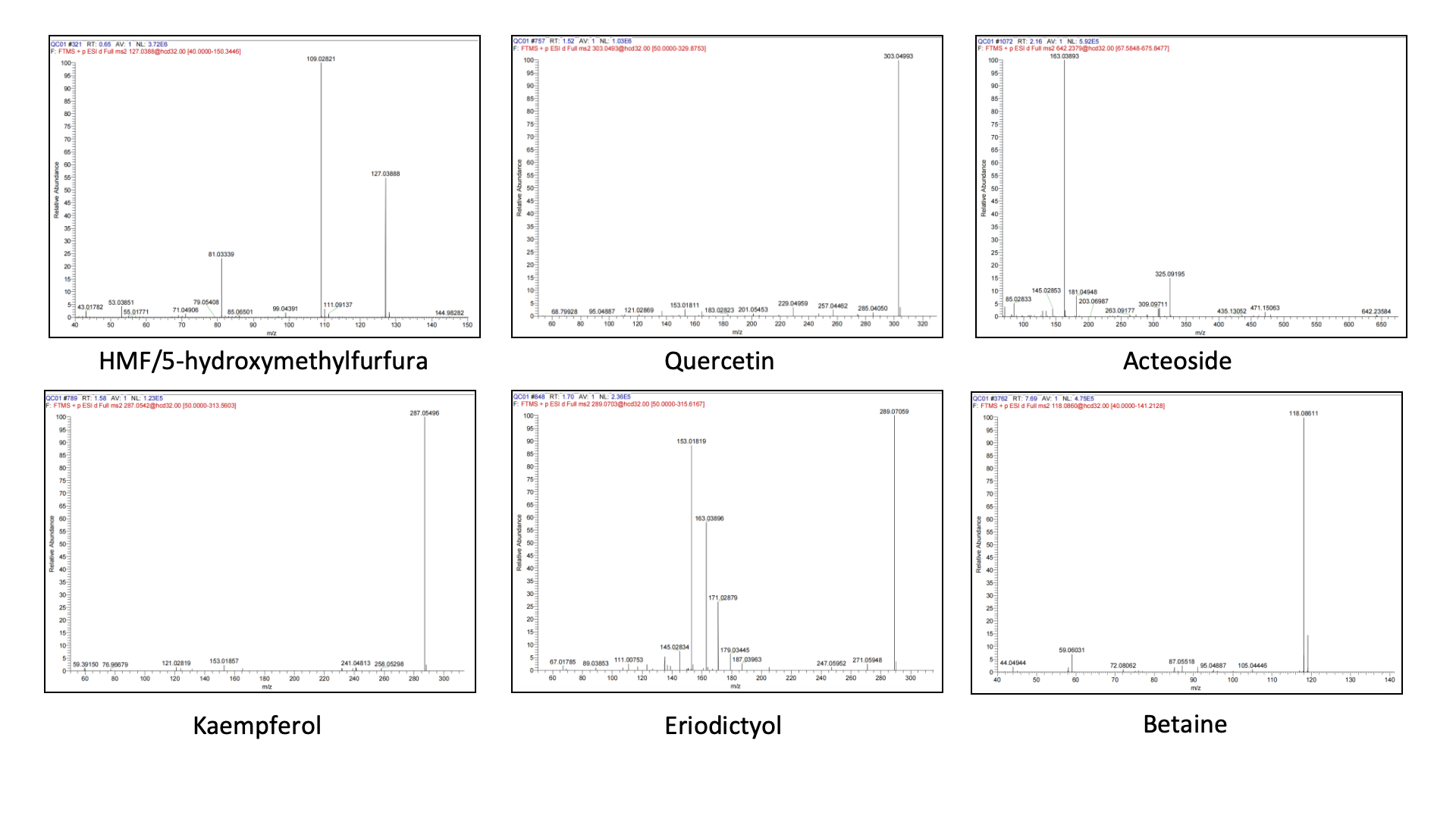
**

**
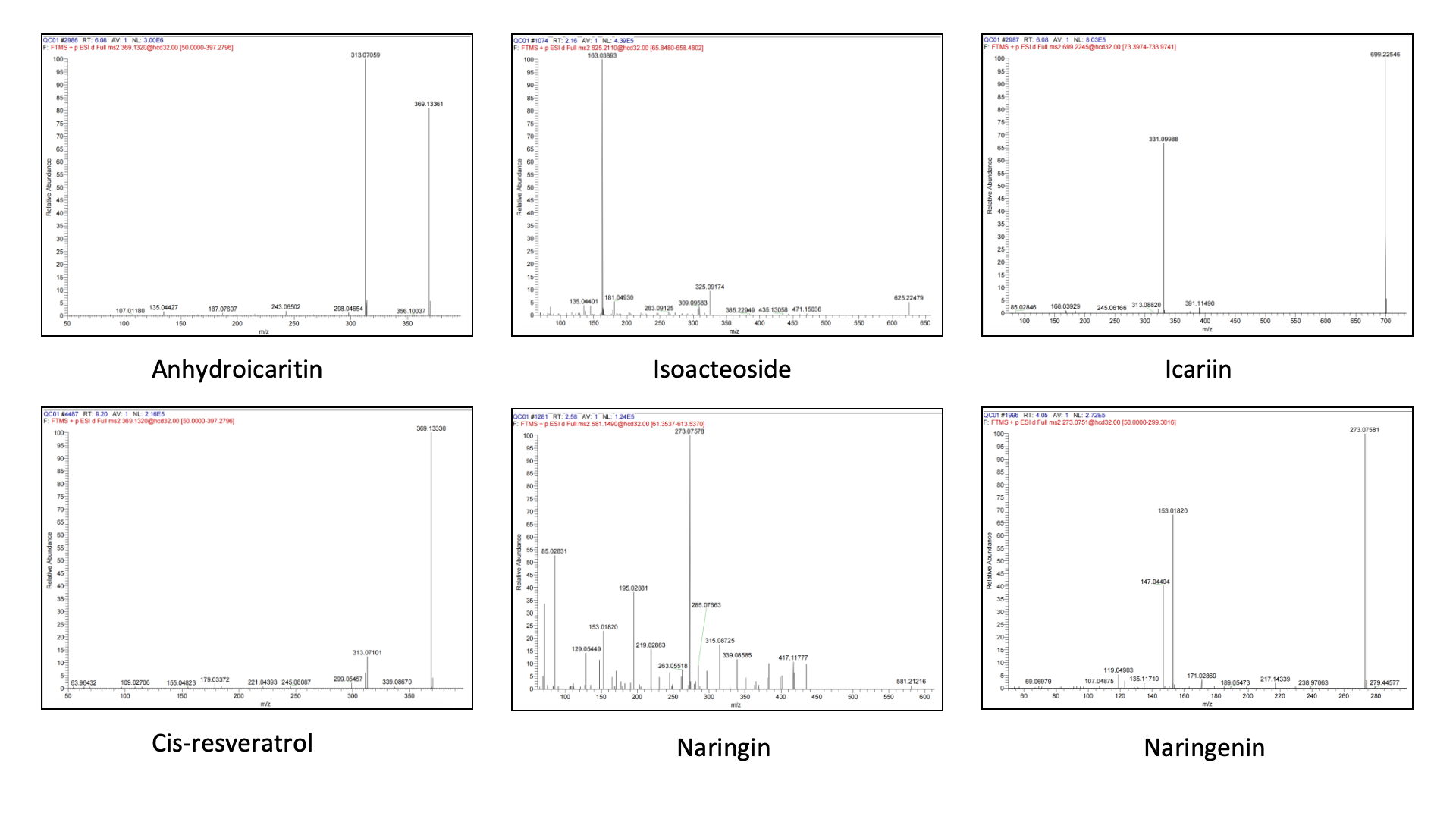
**
